# Cross-Coupling Reaction with Lithium Methyltriolborate

**DOI:** 10.3390/molecules18010430

**Published:** 2012-12-28

**Authors:** Yasunori Yamamoto, Kazuya Ikizakura, Hajime Ito, Norio Miyaura

**Affiliations:** 1 Frontier Chemistry Center, Faculty of Engineering, Hokkaido University, Sapporo 060-8628, Japan; 2 Division of Chemical Process Engineering, Faculty of Engineering, Hokkaido University, Sapporo 060-8628, Japan

**Keywords:** cross-coupling reaction, palladium catalyst, methyltrioolborate

## Abstract

We newly developed lithium methyltriolborate as an air-stable white solid that is convenient to handle. The good performance of this triolborate for metal-catalyzed bond-forming reactions was demonstrated in palladium-catalyzed cross-coupling reactions with haloarenes. Cross-coupling reaction of [MeB(OCH_2_)_3_CCH_3_]Li with aryl halides occurred in the presence of Pd(OAc)_2_/RuPhos complex in refluxing MeOH/H_2_O and the absence of bases.

## 1. Introduction

Over the past three decades, it has become increasingly clear that organoboron compounds are valuable reagents capable of undergoing many catalytic C-C bond formations in organic synthesis [1,2,3,4,5,6]. Boronic acids are convenient reagents that are generally thermally stable and are inert to water and oxygen, and it is easy to remove the inorganic by-products from the reaction mixture, making the reactions suitable for industrial processes. Since the first report in 1986 of the cross-coupling reaction between alkylboron reagents and aryl and alkenyl halides in the presence of a palladium catalyst and a base [7], *B*-alkyl cross-coupling has been frequently used in organic synthesis. Classically, alkylboron reagents have been synthesized from the corresponding alkyllithium or alkylmagnesium compounds by transmetalation with trialkoxyboranes [8]. Similarly, organometallic reagents were trapped with 9-methoxy-9-borabicyclo[3.3.1]nonane (*B*-MeO-9-BBN) to produce the corresponding alkylborinate complexes [9]. Primary alkylboron reagents are easily synthesized by hydroboration of terminal alkenes in a highly chemo-, regio-, and stereoselective manner. Methylboronic acid, methylboroxine [10,11,12,13,14,15,16], and *B*-methyl-9-borabicylco[3.3.1]nonane (*B*-Me-9-BBN) [17,18] can also be employed as coupling partners. However, coupling of a methyl group with various organic halides is less than ideal. Boronic acids are sometimes difficult to purify due to the lack of crystallization or the formation of trimeric cyclic anhydrides (boroxines). For this reason, determination of the stoichiometry of the boronic acid to be used in the reaction is difficult. In addition, cross-coupling of alkylboronic acids is complicated by protodeboronation and, as a result, excess boronic acids are used in the reaction for complete consumption of electrophiles. A recent advance is the use of methylboron reagents, such as MeLi/*B*-MeO-9-BBN [19], 10-methyl-9-oxa-10-borabicyclo[3.3.1]decane [20,21] and MeBF_3_K [22,23,24,25,26,27], for methylation of aryl compounds. However, the use of large amounts of a base, especially a strong base, may be a major limitation for these applications [28]. The development of an efficient, mild and operationally simple catalyst system that does not require the use of large amounts of a base remains a challenge and has becomes an urgent issue.

Recently, we have developed aryltriolborates [ArB(OCH_2_)_3_CCH_3_]M (M = Li, Na, K, and NBu_4_), that have good stability in air and water and high solubility in organic solvents and that undergo very smooth transmetalation to various transition metal complexes [29,30]. High performance for bond-forming reactions was demonstrated in palladium-catalyzed cross-coupling reactions [29,30,31,32,33,34,35], copper-catalyzed *N*-arylation [36] and rhodium-catalyzed asymmetric addition reactions [37,38,39]. We describe herein lithium methyltriolborate that is exceptionally stable in air and water. We also demonstrate the high transmetalation efficiency of triolborate in palladium-catalyzed C-C bond-forming reaction.

## 2. Results and Discussion

**Scheme 1:**
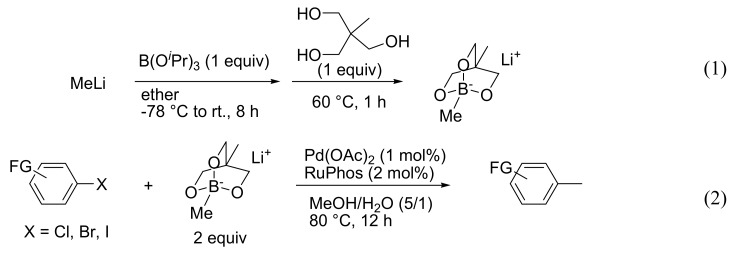
Synthesis of lithium methyltriolborate.

We developed a method for synthesis of lithium methyltriolborate. It was synthesized by methylation of B(O*^i^*Pr)_3_ with MeLi followed by removal of i-PrOH through ester exchange with 1,1,1-tris(hydroxymethyl)ethane (Scheme 1). By using this protocol, [MeB(OCH_2_)_3_CCH_3_]Li was obtained in high yield as an air-stable white solid (97%). Triolborate is a bench-stable ate-complex that can be handled and stored without special precautions.

Next, we chose 4-bromobiphenyl to examine its efficiency toward cross-coupling reaction. The yields were highly sensitive to palladium complexes and phosphine ligands in the cross-coupling reaction between 4-bromobiphenyl and lithium methyltriolborate (Table 1).

When Pd(dba)_2_ was used, the yield was 35% (entry 1). The use of Pd(OAc)_2_ gave the best results (entry 6), but palladium chloride complexes such as PdCl_2_, PdCl_2_(PhCN)_2_, PdCl_2_(PPh_3_)_2_ and PdCl_2_(cod) resulted in yields of 59%, 66%, 67% and 49%, respectively (entries 2–5).

Among phosphine ligands screened for optimizations, RuPhos was found to be best ligand to achieve quantitative yield (entry 7). The use of Brettphos gave a methylation product as in the case of SPhos (entry 8). Other monodentate ligands such as Johnphos, XPhos and PCy_3_ resulted in low yields (entries 9–12). Next, we screened bidentate ligands, and coupling products were obtained in moderate yields (entries 13–17). Furthermore, we optimized the reaction conditions (Table 2). The reaction proceeded smoothly in aqueous MeOH but was very slow in other solvents, such as aqueous THF, toluene, dioxane and DMF (Table 2, entries 1–5). In addition, only water was not effective (entry 8). By further investigations of reaction time (entries 1 and 9–11), amounts of Pd(OAc)_2_ and RuPhos (entries 1, 12, and 13) and temperature (entries 1 and 14), a methylated product was finally obtained in 94% yield using 1 mol% Pd(OAc)_2_/2 mol% RuPhos with MeOH/H_2_O as a solvent at 80 °C for 12 h (entry 13). The yields were low when 1.1 or 1.3 equivalents of boronic acid were used (52% or 83%), but they were increased to practical levels in the presence of 1.5–2.0 equivalents of boronic acid.

### Scope and Limitation

Under the optimized reaction conditions, the scope for representative aryl halides is summarized in Table 3. Quantitative conversions resulting in over 80% yields were easily realized at 80 °C in the presence of Pd(OAc)_2_ (1 mol%) and RuPhos (2 mol%). 2-Naphthyliodide showed a slight decrease in reactivity compared to the corresponding bromide and chloride (entries 6–8). It was also interesting that the steric hindrance of *ortho*-substituents did not affect the yields (entries 10–12). 

The use of 1-bromo-2-methoxynaphthalene, 1-bromo-2-methylnaphthalene and 2-bromo-1,3,5-trimethylbenzene resulted in yields of 96%, 88% and 86%, respectively. The reaction is highly sensitive to electron density of halides. For example, the methylation of furyl and thienyl halides results in low yields (entries 14 and 15).

## 3. Experimental

### 3.1. General

^1^H-NMR spectra were recorded on a JEOL ECX-400 (400 MHz) in CDCl_3_ with tetramethylsilane as an internal standard. Chemical shifts are reported in part per million (ppm), and signal are expressed as singlet (s), doublet (d), triplet (t), quartet (q), multiplet (m), and broad (br). ^13^C-NMR spectra were recorded on a JEOL ECX-400 (100 MHz) in CDCl_3_ (δ_C_ = 77.0) with tetramethylsilane as an internal standard. ^11^B NMR spectra was recorded on a JEOL ECX-400 (128 MHz) with BF_3_·OEt_2_ as an external standard. Chemical shifts are reported in part per million (ppm). Kanto Chemical silica gel 60N (particle size 0.063–0.210 mm) was used for flash column chromatography. All reactions were conducted under an atmosphere of nitrogen. Glassware was oven dried at 130 °C and allowed to cool under a stream of dry nitrogen. All chemicals were purchased from Aldrich, Wako, TCI, or Kanto Chemicals and used as received.

### 3.2. A Preparation of Lithium Methyltriolborate

MeLi (50 mmol) in ether was added to a solution of triisopropoxyborane (50 mmol) in ether (100 mL) at −78 °C. The resulting mixture was stirred for 30 min at −78 °C, then allowed to warm to room temperature and was stirred for 8 h. 1,1,1-tris(hydroxymethyl)ethane (50 mmol) was then added in one portion, and the resulting mixture was stirred at 60 °C for 1 h. The mixture is poured into 1 L of acetone. The solid product is isolated by filtration, washed with acetone and dried under vacuum to afford 7.3 g (97%) of lithium methyltriolborate as a white solid. ^1^H-NMR (DMSO-*d_6_*) δ = 3.40 (s, 6H), 0.37 (s, 3H), −0.75 (s, 3H); ^13^C-NMR (DMSO-*d_6_*) δ = 73.5, 34.5, 16.9, 6.54; ^11^B NMR (DMSO-*d_6_*) δ = 1.44.

### 3.3. General Procedure for Cross-Coupling with Lithium Methyltriolborate

Palladium acetate (1 mol%) and RuPhos (2 mol%) were placed in a flask under an atmosphere of nitrogen. MeOH/H_2_O (2.5 mL/0.5 mL) was added, and then the mixture was stirred for 30 min at room temperature. After addition of lithium methyltriolborate (1 mmol) and aryl halide (0.5 mmol), the mixture was heated at 80 °C for 12 h. After cooling to room temperature, the product was extracted with benzene, and dried over anhydrous MgSO_4_. The desired product was purified by column chromatography on silica gel.

*4-Methylbiphenyl* (entry 1): ^1^H-NMR (CDCl_3_) δ = 7.58 (d, *J* = 7.25 Hz, 2H), 7.49 (d, *J* = 8.15 Hz, 2H), 7.42 (d, *J* = 8.15 Hz, 1H), 7.42 (t, *J* = 7.48 Hz, 2H), 7.32 (t, *J* = 7.48 Hz, 1H), 7.25 (d, *J* = 8.15 Hz, 2H), 2.40 (s, 3H).

*p-Methylacetophenone* (entry 2): ^1^H-NMR (CDCl_3_) δ = 7.82 (d, *J* = 8.15 Hz, 2H), 7.22 (d, *J* = 8.15 Hz, 2H), 2.54 (s, 3H), 2.37 (s, 3H).

*1-Methyl-4-phenoxybenzene* (entry 3): ^1^H-NMR (CDCl_3_) δ = 7.33 (t, *J* = 8.07 Hz, 2H), 7.16 (d, *J* = 8.25 Hz, 2H), 7.09 (t, *J* = 7.53 Hz, 1H), 7.01 (d, *J* = 7.89 Hz, 2H), 6.95 (d, *J* = 8.61 Hz, 2H), 2.36 (s, 3H).

*2-Methoxy-6-methylnaphthalene* (entry 4): ^1^H-NMR (CDCl_3_) δ = 7.70 (d, *J* = 9.06 Hz, 2H), 7.59 (s, 1H), 7.34 (dd, *J* = 1.81, 8.61 Hz, 1H), 7.19 (dd, *J* = 2.72, 8.83 Hz, 1H), 7.15 (d, *J* = 2.27 Hz, 1H), 3.94 (s, 3H), 2.53 (s, 3H).

*2,7-Dimethylnaphthalene* (entry 5): ^1^H-NMR (CDCl_3_) δ = 7.76 (d, *J* = 8.61 Hz, 2H), 7.58 (s, 2H), 7.31 (dd, *J* = 1.36, 8.38 Hz, 2H), 2.56 (s, 3H).

*2-Methylnaphthalene* (entries 6–8): ^1^H-NMR (CDCl_3_) δ = 7.82–7.72 (m, 3H), 7.67 (s, 1H), 7.51–7.40 (m, 2H), 7.33 (d, *J* = 8.15 Hz, 1H), 2.57 (s, 3H).

*1-Methyl-4-nitrobenzene* (entry 9): ^1^H-NMR (CDCl_3_) δ = 8.10 (d, *J* = 8.61 Hz, 2H), 7.31 (d, *J* = 8.61 Hz, 2H), 2.45 (s, 3H).

*2-Methoxy-1-methylnaphthalene* (entry 10): ^1^H-NMR (CDCl_3_) δ = 8.03 (d, *J* = 7.89 Hz, 1H), 7.86 (d, *J* = 8.25 Hz, 1H), 7.78 (d, *J* = 8.97 Hz, 1H), 7.56 (ddd, *J* = 1.43, 6.82, 8.32 Hz, 1H), 7.43 (ddd, *J* = 1.08, 6.67, 7.44 Hz, 1H), 7.32 (d, *J* = 8.97 Hz, 1H), 3.99 (s, 3H), 2.65 (s, 3H).

*1,2-Dimethylnaphthalene* (entry 11): ^1^H-NMR (CDCl_3_) δ = 8.08 (d, *J* = 8.61 Hz, 1H), 7.85 (d, *J* = 7.89 Hz, 1H), 7.67 (d, *J* = 8.25 Hz, 1H), 7.54 (ddd, *J* = 1.43, 6.82, 8.34 Hz, 1H), 7.46 (t, *J* = 6.82 Hz, 1H), 7.35 (d, *J* = 8.25 Hz, 1H), 2.65 (s, 3H), 2.54 (s, 3H).

*1,2,3,5-Tetramethylbenzene* (entry 12): ^1^H-NMR (CDCl_3_) δ = 6.88 (s, 2H), 2.29 (s, 9H), 2.18 (s, 3H).

*2′-Methylbiphenyl-4-carbonitrile* (entry 13): ^1^H-NMR (CDCl_3_) δ = 7.72–7.68 (m, *3*H), 7.44–7.41 (m, 2H), 7.32–7.25 (m, 2H), 7.19 (d, *J* = 7.17 Hz, 1H), 2.26 (s, 3H).

*Methyl 3-methylbenzoate* (entry 14): ^1^H-NMR (CDCl_3_) δ = 7.85–7.82 (m, 2H), 7.36–7.29 (m, 2H), 3.89 (s, 3H), 2.38 (s, 3H).

*Methyl 5-methylfuran-2-carboxylate (entry 15)*: ^1^H-NMR (CDCl_3_) δ = 7.05 (d, *J* = 3.59 Hz, 1H), 6.08 (d, *J* = 3.23 Hz, 1H), 2.34 (s, 3H).

*2-Acetyl-5-methylthiophene* (entry 16): ^1^H-NMR (CDCl_3_) δ = 7.48 (d, *J* = 3.59 Hz, 1H), 6.76 (dd, *J* = 1.08, 3.77 Hz, 1H), 2.50 (s, 3H), 2.41 (s, 3H).

## 4. Conclusions

In summary, we have demonstrated the efficiency of lithium methyltriolborate for methylation of aryl halides. This borate showed several advantages over boronic acid, including high nucleophilicity of methyl groups for smooth transmetalation to a palladium catalyst. Since the use of a base is avoided, a variety of functional groups may be accommodated in this reaction system.

## Figures and Tables

**Table 1:** Effect of ligands *^a^*. 
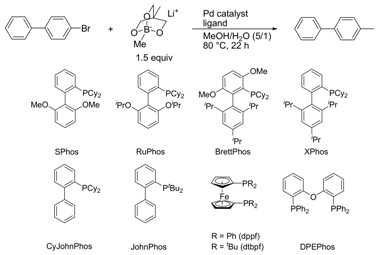

Entry	Pd catalyst (mol%)	Ligand (mol%)	Yield (%) *^b^*
1	Pd(dba)_2_ (3)	SPhos (6)	35
2	PdCl_2_ (3)	SPhos (6)	59
3	PdCl_2_(PPh_3_)_2_ (3)	SPhos (6)	67
4	PdCl_2_(PhCN)_2_ (3)	SPhos (6)	66
5	PdCl_2_(cod) (3)	SPhos (6)	49
6	Pd(OAc)_2_ (3)	SPhos (6)	71
**7**	**Pd(OAc)_2_ (3)**	**RuPhos (6)**	**>99**
8	Pd(OAc)_2_ (3)	BrettPhos (6)	70
9	Pd(OAc)_2_ (3)	XPhos (6)	6
10	Pd(OAc)_2_ (3)	CyJohnPhos (6)	22
11	Pd(OAc)_2_ (3)	JohnPhos (6)	3
12	Pd(OAc)_2_ (3)	PCy_3_ (6)	6
13	Pd(OAc)_2_ (3)	dppp (3)	47
14	Pd(OAc)_2_ (3)	dppb (3)	59
15	Pd(OAc)_2_ (3)	dppf (3)	45
16	Pd(OAc)_2_ (3)	dtbpf (3)	21
17	Pd(OAc)_2_ (3)	DPEphos (3)	44
18	Pd(OAc)_2_ (3)	none	6

*^a^* Reaction conditions: A mixture of 4-bromobiphenyl (1 equiv), lithium methylborate (2 equiv), palladium catalyst (3 mol%) and ligand (3 or 6 mol%) in MeOH/H_2_O (2.5 mL/0.5 mL) at 80 °C for 22 h; *^b^* GC yield.

**Table 2:** Optimaization of methylation by lithium methyltriolborate *^a^*. 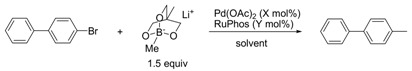

Entry	Solvent	X (mol%)	Y (mol%)	Time (h)	Temp. (°C)	Yield (%) *^b^*
1	MeOH/H_2_O (5/1)	3	6	22	80	>99
2	THF/H_2_O (5/1)	3	6	22	80	35
3	1,4-dioxane/H_2_O (5/1)	3	6	22	80	48
4	toluene/H_2_O (5/1)	3	6	22	80	17
5	DMF/H_2_O (5/1)	3	6	22	80	63
6	MeOH	3	6	22	80	80
7	EtOH	3	6	22	80	79
8	H_2_O	3	6	22	80	9
9	MeOH/H_2_O (5/1)	3	6	12	80	>99
10	MeOH/H_2_O (5/1)	3	6	6	80	86
11	MeOH/H_2_O (5/1)	3	6	1	80	61
12	MeOH/H_2_O (5/1)	2	4	12	80	>99
13	MeOH/H_2_O (5/1)	1	2	12	80	>99 (94 *^c^*)
14	MeOH/H_2_O (5/1)	1	2	12	60	90

*^a^* Reaction conditions: A mixture of 4-bromobiphenyl (1 equiv), lithium methylborate (2 equiv), Pd(OAc)_2_ and RuPhos; *^b^* GC yield; *^c^* Isolated yield.

**Table 3:** Cross-coupling between lithium methyltriolborate and aryl halides *^a^*.
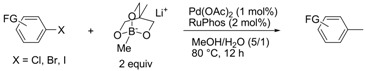

Entry	Substrate	Yield (%) *^b^*	Entry	Substrate	Yield (%) *^b^*
1	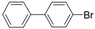	94 *^c^*	9	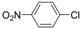	50 *^e^*
2	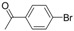	95	10	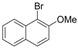	96
3	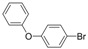	81	11	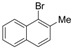	88
4	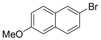	>99	12	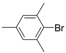	86
5	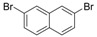	88 *^d^*	13	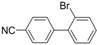	88
6	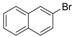	96	14	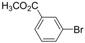	72
7	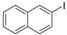	77	15	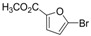	66
8	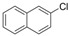	94	16	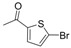	64

*^a^* Reaction conditions: A mixture of aryl halides (1 equiv), lithium methylborate (2 equiv), Pd(OAc)_2_ (1 mol%) and RuPhos (2 mol%) in MeOH/H_2_O (2.5 mL/0.5 mL) was stirred at 80 °C for 12 h; *^b^* Isolated yield; *^c^* Lithium methyltriolborate (1.5 eq.) was used; *^d^* Lithium methyltriolborate (4 eq.), Pd(OAc)_2_ (2 mol%) and RuPhos (4 mol%) were used; *^e^* 4-methoxy-nitrobenzene (26%) was formed.
